# Do horizontal propulsive forces influence the nonlinear structure of locomotion?

**DOI:** 10.1186/1743-0003-4-30

**Published:** 2007-08-15

**Authors:** Max J Kurz, Nicholas Stergiou

**Affiliations:** 1 Laboratory of Integrated Physiology, University of Houston, Department of Health and Human Performance, Houston, Texas, USA; 2 HPER Biomechanics Laboratory, University of Nebraska at Omaha, School of HPER, Omaha, Nebraska, USA

## Abstract

**Background:**

Several investigations have suggested that changes in the nonlinear gait dynamics are related to the neural control of locomotion. However, no investigations have provided insight on how neural control of the locomotive pattern may be directly reflected in changes in the nonlinear gait dynamics. Our simulations with a passive dynamic walking model predicted that toe-off impulses that assist the forward motion of the center of mass influence the nonlinear gait dynamics. Here we tested this prediction in humans as they walked on the treadmill while the forward progression of the center of mass was assisted by a custom built mechanical horizontal actuator.

**Methods:**

Nineteen participants walked for two minutes on a motorized treadmill as a horizontal actuator assisted the forward translation of the center of mass during the stance phase. All subjects walked at a self-select speed that had a medium-high velocity. The actuator provided assistive forces equal to 0, 3, 6 and 9 percent of the participant's body weight. The largest Lyapunov exponent, which measures the nonlinear structure, was calculated for the hip, knee and ankle joint time series. A repeated measures one-way analysis of variance with a t-test post hoc was used to determine significant differences in the nonlinear gait dynamics.

**Results:**

The magnitude of the largest Lyapunov exponent systematically increased as the percent assistance provided by the mechanical actuator was increased.

**Conclusion:**

These results support our model's prediction that control of the forward progression of the center of mass influences the nonlinear gait dynamics. The inability to control the forward progression of the center of mass during the stance phase may be the reason the nonlinear gait dynamics are altered in pathological populations. However, these conclusions need to be further explored at a range of walking speeds.

## Background

Human and animal locomotion is typically described as having a periodic movement pattern. For example, it can be readily observed that the legs oscillate to-and-fro with a limit cycle behavior that is similar to the pendulum motions of a clock [1,2]. Any variations from this periodic pattern have traditionally been considered to be "noise" within the neuromuscular system [3,4]. However, recent investigations have confirmed that the step-to-step variations that are present in gait may not be strictly noise. Rather these variations may have a deterministic structure [3,5-12]. Several authors have noted that the structure of the nonlinear gait dynamics is influenced by the health of the neuromuscular system. These results imply that the observed changes in the nonlinear gait dynamics may be related to the organization of the nervous system for functional and stable gait [3,5-9]. Although this seems plausible, no efforts have been made to explore what neural control strategies can govern the nonlinear gait dynamics. Such insight may lead to new clinical methods for assessing the health of the neuromuscular system, and may lead to new metrics that can be used to guide the rehabilitation of the neuromuscular system.

The control of human locomotion can be globally divided into the stance and swing phases. A major determinant of the stance phase is the ability of the neuromuscular system to redirect the center of mass forward and over the support limb for each step of the gait cycle [13,14]. Proper neuromuscular control of the center of mass allows for the locomotive system to take advantage of the energy exchange that is associated with the inverted pendulum dynamics [13]. Possibly the neural control strategies that dictate the forward progression of the center of mass may also influence the nonlinear structure of human locomotion. In this investigation, we explored if the control of the forward progression of the center of mass during the stance phase may govern the nonlinear gait dynamics.

**Figure 1:**
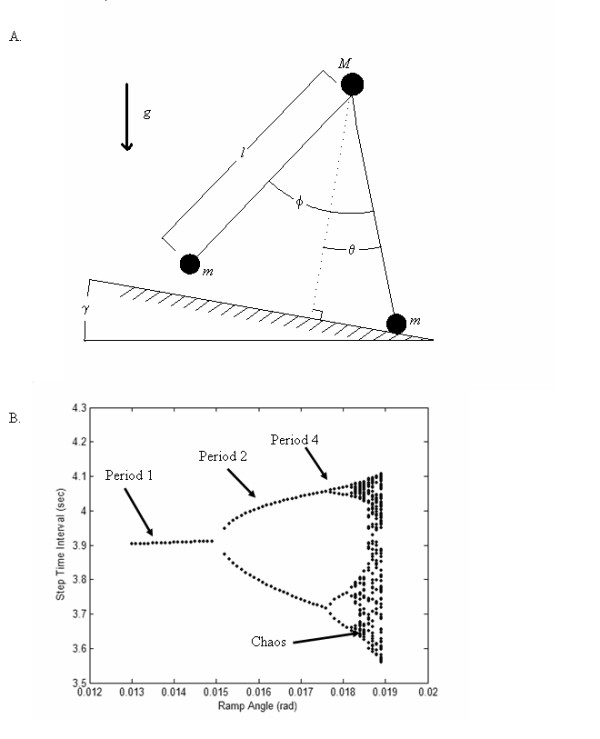
(A) Passive dynamic walking model where *φ *is the angle of the swing leg, *θ *is the angle of the stance leg, m is the point mass at the respective foot, M is the mass at the hip, *γ *is the angle of inclination of the supporting surface, and g is gravity. Both legs are of length ℓ. (B) Cascade of bifurcations in the step time interval of the passive dynamic walking model with no toe-off impulse applied (*e.g*., J = 0). A period-1 gait means the model selects the same step-time interval for gait pattern, a period-2 means that the model alternates between two different step-time intervals, and a period-4 means that the model uses four different step-time intervals. Nonlinear gait patterns that are chaotic are found when the ramp angle is between 0.01839 radians and 0.0189 radians when J = 0. No stable gaits are present if the ramp angle is greater than 0.019 radians [19].

**Figure 2:**
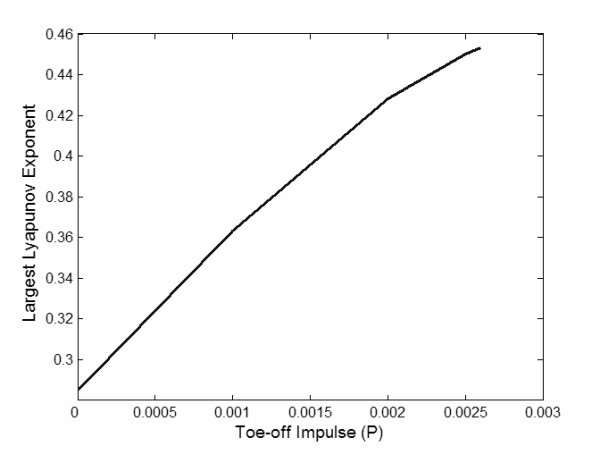
Largest Lyapunov exponent values as the toe-off impulse (J) was increased in each simulation. The ramp angle remained fixed at 0.0185 radians for all simulations. The simulations predict that the magnitude of the largest Lyapunov exponent will increase as the toe-off impulse is increased and assists the forward progression of the center of mass. These simulations indicate that toe-off impulses can be used to control the structure of the chaotic gait pattern.

Much of the insights on the origin of the nonlinear dynamics of physical and biological systems have come from the analysis of simplified mathematical models that are sufficiently close to the behavior of the real system [15]. Full and Koditschek [16] referred to such simple models as templates. A template for locomotion has all the joint complexities, muscles and neurons of the locomotive system removed [16]. Recently, passive dynamic walking models have proven to be a viable template for the exploration of nonlinear gait dynamics [17-21]. These models consist of an inverted double pendulum system that captures the dynamics of the swing and stance phase (Figure 1A). Energy for the locomotive pattern comes from a slightly sloped walking surface. Compared to other walking models, this model is unique because as the walking surface angle increases, there is a cascade of bifurcations in the model's gait pattern that eventually converges to a nonlinear gait pattern that is similar to humans (Figure 1B) [19]. To gain insight on how neural control strategies influence the nonlinear gait dynamics, we modified the governing equations of the passive dynamic walking model to include an instantaneous toe-off impulse (J) that assisted the forward motion of the center of mass. Our simulations indicated that toe-off impulses influenced the bifurcations and nonlinear structure of the passive dynamic walking model's gait. Changes in the nonlinear structure were quantified by calculating the largest Lyapunov exponent for the model's gait. Our simulations indicated that the magnitude of the largest Lyapunov exponent linearly increased as the amount of assistance provided by the toe-off impulse (J) was increased in each simulation (Figure 2). These simulations predict that neural control of the forward progression of the center of mass during the stance phase influences the nonlinear structure of human locomotion.

To test the prediction of our model, we built a mechanical horizontal actuator that assisted the forward motion of the center of mass during the stance phase. Based on the results of our simulations, we hypothesized that the magnitude of the largest Lyapunov exponent is dependent on neural control of the forward progression of the center of mass during the stance phase of gait.

## Methods

### Nonlinear Analysis Techniques

The following analysis techniques were used to quantify the nonlinear structure of the gait patterns of the computer simulations, and the complementary human experiment conducted in this investigation.

From the original time series (*i.e*., knee angle), the state space was reconstructed based on Taken's embedding theorem [22,23]. The reconstruction process involved creating time-lagged copies of the original time series. Equation 1 presents the reconstructed state vector where **y**(t) was the reconstructed state vector, x(t) was the original time series data, and x(t-T_i_) was time delay copies of x(t).

**y**(t) = [x(t), x(t-T_1_), x(t-T_2_),...].

The time delay (T_i_) for creating the state vector was determined by estimating when information about the state of the dynamic system at x(t) was different from the information contained in its time-delayed copy using an average mutual information algorithm [22]. Equation 2 presents the average mutual information algorithm used in this investigation where T was the time delay, x(t) was the original data, x(t+T) was the time delay data, P(x(t), x(t+T)) was the joint probability for measurement of x(t) and x(t+T), P(x(t)) was the probability for measurement of x(t), and P(x(t+T)) was the probability for measurement of x(t+T).

Ix(t),x(t+T)=∑P(x(t),x(t+T))log⁡2[P(x(t),x(t+T))P(x(t))P(x(t+T))].
 MathType@MTEF@5@5@+=feaafiart1ev1aaatCvAUfKttLearuWrP9MDH5MBPbIqV92AaeXatLxBI9gBaebbnrfifHhDYfgasaacH8akY=wiFfYdH8Gipec8Eeeu0xXdbba9frFj0=OqFfea0dXdd9vqai=hGuQ8kuc9pgc9s8qqaq=dirpe0xb9q8qiLsFr0=vr0=vr0dc8meaabaqaciaacaGaaeqabaqabeGadaaakeaacqWGjbqsdaWgaaWcbaGaemiEaGNaeiikaGIaemiDaqNaeiykaKIaeiilaWIaemiEaGNaeiikaGIaemiDaqNaey4kaSIaemivaqLaeiykaKcabeaakiabg2da9maaqaeabaGaemiuaaLaeiikaGIaemiEaGNaeiikaGIaemiDaqNaeiykaKcaleqabeqdcqGHris5aOGaeiilaWIaemiEaGNaeiikaGIaemiDaqNaey4kaSIaemivaqLaeiykaKIaeiykaKIagiiBaWMaei4Ba8Maei4zaC2aaSbaaSqaaiabikdaYaqabaGcdaWadaqaamaalaaabaGaemiuaaLaeiikaGIaemiEaGNaeiikaGIaemiDaqNaeiykaKIaeiilaWIaemiEaGNaeiikaGIaemiDaqNaey4kaSIaemivaqLaeiykaKIaeiykaKcabaGaemiuaaLaeiikaGIaemiEaGNaeiikaGIaemiDaqNaeiykaKIaeiykaKIaemiuaaLaeiikaGIaemiEaGNaeiikaGIaemiDaqNaey4kaSIaemivaqLaeiykaKIaeiykaKcaaaGaay5waiaaw2faaiabc6caUaaa@7485@

**Figure 3:**
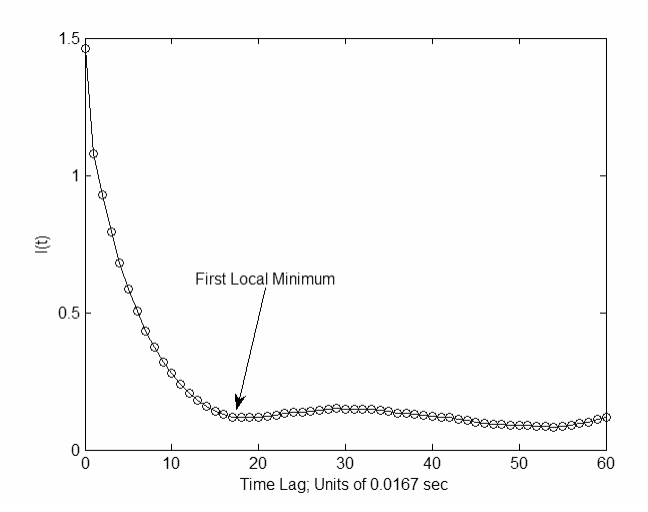
Average mutual information algorithm is used to locate the time lag at the first local minimum.

Average mutual information was iteratively calculated for various time delays, and the selected time delay was the first local minimum of the iterative process (Figure 3) [22,23]. This selection was based on previous investigations that have determined that the time delay at the first local minimum contains sufficient information about the dynamics of the system to reconstruct the state vector [22].

The number of embedding dimensions of the data time series was calculated to unfold the dynamics of the system in an appropriate state space. An inappropriate number of embedding dimensions may result in a projection of the dynamics of the system that has orbital crossings in the state space that are due to false neighbors, and not the actual dynamics of the system [22,23]. To unfold the state space, we systematically inspected x(t), and its neighbors in various dimensions (*e.g*., dimension = 1, 2, 3,...etc.). The appropriate embedding dimension was identified when the neighbors of the x(t) stopped being un-projected by the addition of further dimensions of the state vector. For example, the global false nearest neighbors algorithm compares the points in the attractor at a given dimension d_E_

**y**(t) = [x(t), x(t + T), x(t + 2T), ... x(t + (d_E_-1) T)].

**Figure 4:**
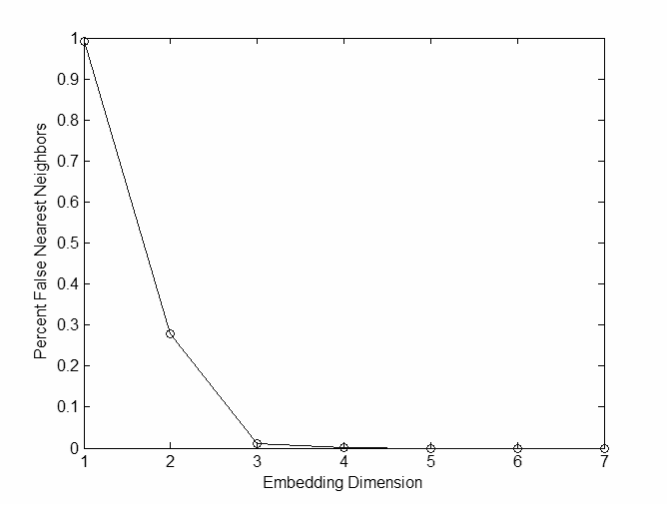
Global false nearest neighbor algorithm is used to determine the embedding dimension where the percent of global false nearest neighbors drops to zero percent.

**y**^NN^(t) = [x^NN^(t), x^NN^(t + T), x^NN^(t + 2T), ... x^NN^(t + (d_E_-1) T)].

where **y**(t) is the current point being considered, and **y**^NN^(t) is the nearest neighbor. If the distance between the points at the next dimension (e.g., d_E+1_) is greater than the distance calculated at the current dimension (e.g., d_E_), then the point is considered a false neighbor and further embeddings are necessary to unfold the attractor. The percentage of false nearest neighbors was calculated at higher dimensions until the percent nearest neighbors dropped to zero (Figure 4). The embedding dimension that had zero percent false nearest neighbors was used to re-construct the attractor in an appropriate state space. Equation 5 presents a reconstructed state vector where d_E _was the number of embedding dimensions, **y**(t) was the d_E_-1 dimensional state vector, x(t) was the original data, and T was the time delay.

**y**(t) = [x(t), x(t + T), x(t + 2T), ... x(t + (d_E_-1) T)].

The Applied Nonlinear Dynamics software was used to calculate the time lags and embedding dimensions for the computer simulations and complementary human experiments.

**Figure 5:**
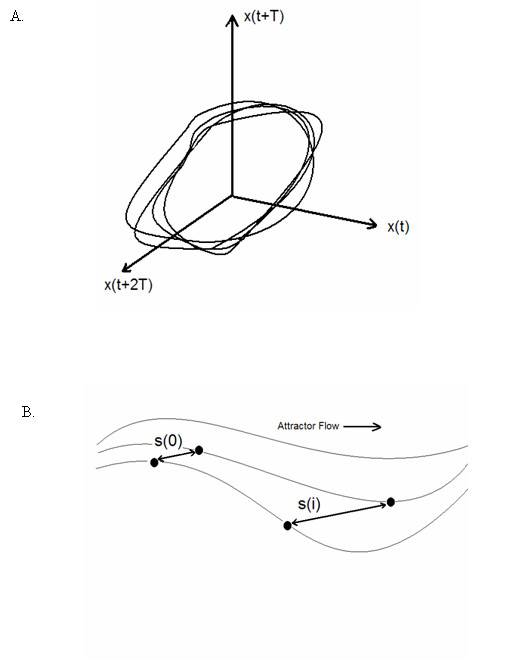
(A) Hypothetical reconstructed attractor, (B) zoomed-inwindow of the reconstructed attractor where s(0) is the initial Euclidean distance between two neighboring points in the attractor and s(i) is the Euclidean distance between the two points i times later. The larger the divergance of the two points over time, the larger the Lyapunov exponent value.

The largest Lyapunov exponent was calculated to determine the nonlinear structure of the reconstructed attractor. Lyapunov exponents quantify the average rate of separation or divergence of points in the attractor over time [22,23]. Figure 5A presents a hypothetical reconstructed attractor, and Figure 5B is a zoomed-in portion of the reconstructed attractor. Figure 5B depicts two neighboring points in the reconstructed attractor that are separated by an initial distance of s(0). As time evolves, the two points diverge rapidly and are separated by a distance of s(i). The Lyapunov exponent is a measure of the logarithmic divergence of the pairs of neighboring points in the attractor over time. The larger the Lyapunov exponent, the greater the divergence in the reconstructed attractor. For the simulations and complementary human experiments conducted here, we used the *Chaos Data Analyzer *(American Institute of Physics) to numerically calculate the largest Lyapunov exponent.

### Walking Model

A simplified passive dynamic walking model was used to initially predict the effects of a toe-off impulse on the nonlinear gait dynamics (Figure 1A). The equations of motion for the model were as follows:

θ¨(t)−sin⁡(θ(t)−γ)=0
 MathType@MTEF@5@5@+=feaafiart1ev1aaatCvAUfKttLearuWrP9MDH5MBPbIqV92AaeXatLxBI9gBaebbnrfifHhDYfgasaacH8akY=wiFfYdH8Gipec8Eeeu0xXdbba9frFj0=OqFfea0dXdd9vqai=hGuQ8kuc9pgc9s8qqaq=dirpe0xb9q8qiLsFr0=vr0=vr0dc8meaabaqaciaacaGaaeqabaqabeGadaaakeaaiiGacuWF4oqCgaWaaiabcIcaOiabdsha0jabcMcaPiabgkHiTiGbcohaZjabcMgaPjabc6gaUjabcIcaOiab=H7aXjabcIcaOiabdsha0jabcMcaPiabgkHiTiab=n7aNjabcMcaPiabg2da9iabicdaWaaa@41BA@

θ¨(t)−φ¨(t)+θ˙(t)2sin⁡(φ(t))−cos⁡(θ(t)−γ)sin⁡(φ(t))=0
 MathType@MTEF@5@5@+=feaafiart1ev1aaatCvAUfKttLearuWrP9MDH5MBPbIqV92AaeXatLxBI9gBaebbnrfifHhDYfgasaacH8akY=wiFfYdH8Gipec8Eeeu0xXdbba9frFj0=OqFfea0dXdd9vqai=hGuQ8kuc9pgc9s8qqaq=dirpe0xb9q8qiLsFr0=vr0=vr0dc8meaabaqaciaacaGaaeqabaqabeGadaaakeaaiiGacuWF4oqCgaWaaiabcIcaOiabdsha0jabcMcaPiabgkHiTiqb=z8aMzaadaGaeiikaGIaemiDaqNaeiykaKIaey4kaSIaf8hUdeNbaiaacqGGOaakcqWG0baDcqGGPaqkdaahaaWcbeqaaiabikdaYaaakiGbcohaZjabcMgaPjabc6gaUjabcIcaOiab=z8aMjabcIcaOiabdsha0jabcMcaPiabcMcaPiabgkHiTiGbcogaJjabc+gaVjabcohaZjabcIcaOiab=H7aXjabcIcaOiabdsha0jabcMcaPiabgkHiTiab=n7aNjabcMcaPiGbcohaZjabcMgaPjabc6gaUjabcIcaOiab=z8aMjabcIcaOiabdsha0jabcMcaPiabcMcaPiabg2da9iabicdaWaaa@63D4@

where *θ *was the angle of the stance leg, *φ *was the angle of the swing leg and θ˙
 MathType@MTEF@5@5@+=feaafiart1ev1aaatCvAUfKttLearuWrP9MDH5MBPbIqV92AaeXatLxBI9gBaebbnrfifHhDYfgasaacH8akY=wiFfYdH8Gipec8Eeeu0xXdbba9frFj0=OqFfea0dXdd9vqai=hGuQ8kuc9pgc9s8qqaq=dirpe0xb9q8qiLsFr0=vr0=vr0dc8meaabaqaciaacaGaaeqabaqabeGadaaakeaaiiGacuWF4oqCgaGaaaaa@2E72@, θ¨
 MathType@MTEF@5@5@+=feaafiart1ev1aaatCvAUfKttLearuWrP9MDH5MBPbIqV92AaeXatLxBI9gBaebbnrfifHhDYfgasaacH8akY=wiFfYdH8Gipec8Eeeu0xXdbba9frFj0=OqFfea0dXdd9vqai=hGuQ8kuc9pgc9s8qqaq=dirpe0xb9q8qiLsFr0=vr0=vr0dc8meaabaqaciaacaGaaeqabaqabeGadaaakeaaiiGacuWF4oqCgaWaaaaa@2E73@ and φ¨
 MathType@MTEF@5@5@+=feaafiart1ev1aaatCvAUfKttLearuWrP9MDH5MBPbIqV92AaeXatLxBI9gBaebbnrfifHhDYfgasaacH8akY=wiFfYdH8Gipec8Eeeu0xXdbba9frFj0=OqFfea0dXdd9vqai=hGuQ8kuc9pgc9s8qqaq=dirpe0xb9q8qiLsFr0=vr0=vr0dc8meaabaqaciaacaGaaeqabaqabeGadaaakeaaiiGacuWFgpGzgaWaaaaa@2E76@ were the respective time derivatives, *γ *is the angle of the walking surface, and t is time. Equation 6 represents the stance leg and equation 7 represents the swing leg. Derivations of the equations of motion for the walking model are detailed in Garcia *et al*. [17].

The governing equations were integrated using a modified version of *Matlab's *(MathWorks, Natick, MA) ODE45. The ODE45 was modified to integrate the equations of motion with a tolerance of 10^-11^, and to stop integrating when the angle of the swing leg angle was twice as large as the stance leg angle (Equation 8).

*φ *- 2*θ *= 0

The swing leg became the stance leg and the former stance leg became the swing leg when the conditions presented in equation 8 were satisfied. The control properties of the ankle joint in assisting the forward progression of the center of mass were modeled in the transition equation (Equation 9).

(θθ˙φφ˙)+=(−10000cos⁡2θ00−20000cos⁡2θ(1−cos⁡2θ)00)(θθ˙φφ˙)−+(0sin⁡2θ0(1−cos⁡2θ)sin⁡2θ)J.
 MathType@MTEF@5@5@+=feaafiart1ev1aaatCvAUfKttLearuWrP9MDH5MBPbIqV92AaeXatLxBI9gBaebbnrfifHhDYfgasaacH8akY=wiFfYdH8Gipec8Eeeu0xXdbba9frFj0=OqFfea0dXdd9vqai=hGuQ8kuc9pgc9s8qqaq=dirpe0xb9q8qiLsFr0=vr0=vr0dc8meaabaqaciaacaGaaeqabaqabeGadaaakeaadaqadaqaauaabeqaeeaaaaqaaGGaciab=H7aXbqaaiqb=H7aXzaacaaabaGae8NXdygabaGaf8NXdyMbaiaaaaaacaGLOaGaayzkaaWaaWbaaSqabeaacqGHRaWkaaGccqGH9aqpdaqadaqaauaabeqaeqaaaaaabaGaeyOeI0IaeGymaedabaGaeGimaadabaGaeGimaadabaGaeGimaadabaGaeGimaadabaGagi4yamMaei4Ba8Maei4CamNaeGOmaiJae8hUdehabaGaeGimaadabaGaeGimaadabaGaeyOeI0IaeGOmaidabaGaeGimaadabaGaeGimaadabaGaeGimaadabaGaeGimaadabaGagi4yamMaei4Ba8Maei4CamNaeGOmaiJae8hUdeNaeiikaGIaeGymaeJaeyOeI0Iagi4yamMaei4Ba8Maei4CamNaeGOmaiJae8hUdeNaeiykaKcabaGaeGimaadabaGaeGimaadaaaGaayjkaiaawMcaamaabmaabaqbaeqabqqaaaaabaGae8hUdehabaGaf8hUdeNbaiaaaeaacqWFgpGzaeaacuWFgpGzgaGaaaaaaiaawIcacaGLPaaadaahaaWcbeqaaiabgkHiTaaakiabgUcaRmaabmaabaqbaeqabqqaaaaabaGaeGimaadabaGagi4CamNaeiyAaKMaeiOBa4MaeGOmaiJae8hUdehabaGaeGimaadabaGaeiikaGIaeGymaeJaeyOeI0Iagi4yamMaei4Ba8Maei4CamNaeGOmaiJae8hUdeNaeiykaKIagi4CamNaeiyAaKMaeiOBa4MaeGOmaiJae8hUdehaaaGaayjkaiaawMcaaiabdQeakjabc6caUaaa@87A9@

where "+" indicated the behavior of the model just after heel-contact, "-" indicated the behavior of the model just before heel-contact, and J represents an instantaneous toe-off impulse that is directed toward the center of mass. J was dimensionless and had a normalization factor M(g l)^1/2^. Further details on the derivation of the transition equation are found in Kuo [24]. A toe-off impulse was included in our model because several experimental investigations with humans have demonstrated that the ankle joint is a major contributor for the forward progression of the center of mass during locomotion [25,26].

Analyses of the locomotive patterns of the model were performed from 3,000 footfalls with the first 500 footfalls removed to be certain that the model converged to the given attractor. The step time intervals were used to classify the gait pattern of the walking model. The influence of the toe-off impulse on the nonlinear structure of the model's gait was explored by systematically increasing J in the transition equation while the ramp angle remained constant in the governing equations. For each simulation, the largest Lyapunov exponent was calculated for the respective step time interval time series to determine how the altered toe-off impulses influenced the model's nonlinear gait dynamics using a time lag of one and an embedding dimension of three [19].

### Experimental Procedures

Nineteen subjects (14 Females, 5 Males; Age = 25.89 ± 5 years; Weight = 665.8 ± 79.8 N; Height = 1.68 ± 0.06 m) volunteered to participate in this investigation. All subjects were in good health and free from any musculoskeletal injuries and disorders. The experimental protocol used in this investigation was approved by the University's Internal Review Board and all subjects provided written informed consent. All subjects had treadmill walking experience prior to participating in the experiment.

**Figure 6:**
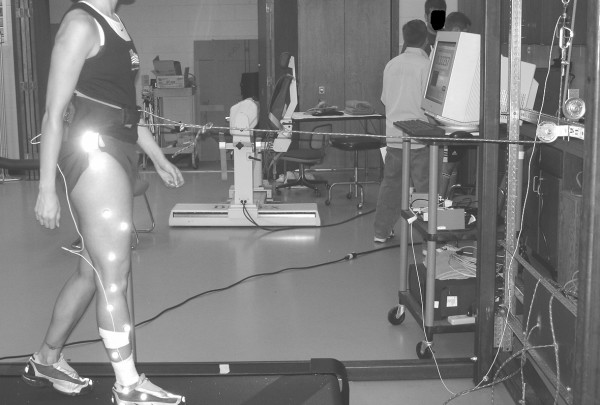
The mechanical horizontal actuator used in this investigation supplied a linear force at the center of mass during the stance phase. The device consisted of a cable-spring pulley system. The magnitude restoring force supplied by the spring during the stance phase was adjusted with a hand winch.

A mechanical horizontal actuator was designed to assist the forward motion of the center of mass during the stance phase (Figure 6). The horizontal actuator applied a linear force at the center of mass of the subject via a cable-spring winch system as the subject walked on a reversible treadmill (Bodygaurd Fitness, St-Georges, Quebec, Canada). Similar actuators have been used to explore the metabolic cost and neural control of locomotion [25,27,28]. Additionally, Gottschall and Kram [25] previously determined that a horizontal mechanical actuator can be use to assist the ankle joint in the forward progression of the center of mass. To set the horizontal force actuator to a specific force value, the subject stood at the middle of the length of the treadmill. The length of the rubber spring was adjusted with a hand winch and the force was measured with a piezoelectric load cell (PCB Piezotronics Inc., Depew, New York) that was in series with the cable-spring-winch system. To ensure that the mechanical horizontal force actuator supplied the same assistance, markers were placed on the treadmill to serve as reminders of the proper position on the treadmill. Additionally, during the data collection, the subjects were coached to maintain their gait between the markers and could view the force value of the load cell on a computer monitor. Subjects initially warmed up and accommodated to the motion of the treadmill for approximately five minutes. This was followed by having the subjects accommodate to walking with the mechanical actuator assisting the forward motion of the center of mass at the experimental levels. The subjects continued to walk on the treadmill until the subject stated that they felt stable walking while attached to the mechanical actuator.

The subjects walked on the treadmill for two minutes at a self-selected pace while the mechanical horizontal actuator assisted the forward motion of the center of mass. The average walking speed that was used for all conditions was 1.01 ± 0.2 ms^-1 ^(Cadence = 116 ± 8 steps/min). The horizontal force actuator supplied a force equal to 0%, 3%, 6% and 9% of the subject's body weight. These percentages were selected based on our pilot data, where we determined that they provided the minimal distortion of the normal gait pattern. A high-speed digital four camera motion capture system (Motion Analysis, Santa Rosa, California) was used to capture the three dimensional positions of reflective markers placed on the lower extremity at 60 Hz. Triangulations of markers were placed on the thigh, shank and foot segments. A standing calibration was used to correct for misalignment of the markers with the local coordinate system of each of the lower extremity segments. This was accomplished by having the subjects stand in a calibration fixture that was aligned with the global reference system. Custom software was used to calculate the three-dimensional segment and joint angles consistent with Vaughan *et al*. [29] from the corrected positions of the segment markers. The joint angle time series were analyzed unfiltered in order to get a more accurate representation of the variability within the system [30]. Previous investigations have indicated that filtering the data may eliminate important information and provide a skewed view of the system's inherent variability [31]. Using the nonlinear analysis techniques discussed in Section A of the methods, the largest Lyapunov exponents for the respective joint angle time series were numerically calculated with an embedding dimension of six.

A one-way analysis of variances (ANOVA) with repeated-measures design was performed for each joint to determine statistical significance between the means of the respective assistance conditions. Furthermore, we used dependent t-tests with a Bonferroni adjustment as a post-hoc test to analyze if the respective assistance conditions were different from the no assistance condition. The alpha level was defined as *P *< 0.05. A linear trend analysis was performed if statistical differences were found. The trend analysis allowed us to infer if the nonlinear structure of the human gait pattern scaled in a similar fashion as the passive dynamic walking model computer simulations.

## Results and Discussion

### Simulation Results

Our simulations indicated that systematic increase in the toe-off impulse (J>0) resulted in the largest Lyapunov exponent to have a greater magnitude. For example, at a ramp angle of 0.0185 radians the largest Lyapunov exponent for the model's nonlinear gait pattern was 0.285 when J = 0. However, if a toe-off impulse was used to assist the forward progression of the center of mass (J = 0.001), the largest Lyapunov exponent of the model's gait pattern increased to a value of 0.363. These results are further detailed in Figure 2 where it is apparent that the largest Lyapunov exponent's magnitude linearly increased as a greater toe-off impulse was used to assist the forward progression of the model's center of mass. Therefore, the simulations predict that an increase in the propulsive forces that govern the forward translation of the center of mass during the stance phase will result in a linear increase in the magnitude of the largest Lyapunov exponent in a human's gait pattern.

### Experimental Results

**Figure 7:**
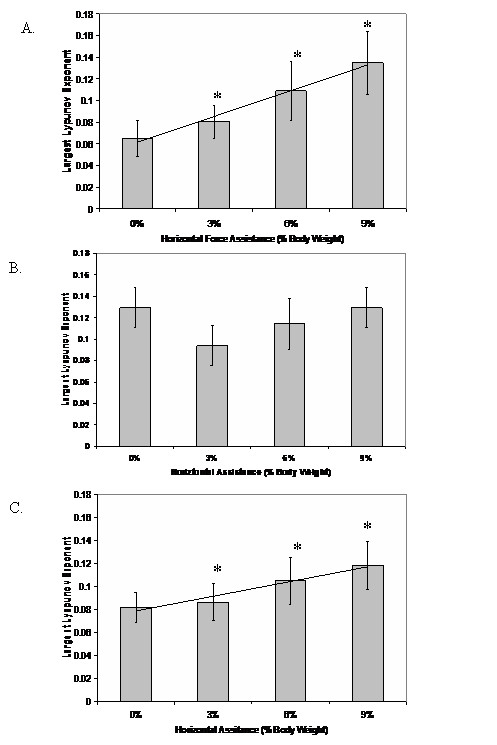
Largest Lyapunov exponent values for the hip (A), knee (B) and ankle (C) joints as the percent of horizontal assistance by the mechanical actuator was increased. The line represents the significant linear trend for the respective horizontal assistance conditions. All of the horizontal assistance conditions for the ankle and hip joints were significantly different (p < 0.05) from the no assistance condition (i.e., 0%). No significant differences were found for the knee joint.

A significant difference was found for the hip (F(3,54) = 134.03, p = 0.0001) and the ankle (F(3,54) = 38.99, p = 0.0001) joint's largest Lyapunov exponent for the horizontal assistance conditions (Figure 7). Post-hoc analysis indicated a significant difference between 0% and all the assistance conditions for the hip and the ankle. No significant differences were found for the knee joint during the horizontal assistance conditions (F(3,54) = 0.605, p = 0.62; Figure 7). There was a significant increasing linear trend for the hip (F(1,18) = 267.16, p = 0.0001) and the ankle (F(1,18) = 146.73, p = 0.0001) joints' largest Lyapunov exponent as the horizontal assistance was increased. These results indicated that the nonlinear structure of the ankle and hip joints' movement patterns were altered as horizontal assistance was increased.

The experimental results are consistent with the hypothesis that the nonlinear structure of gait is dependent on the neural control of the forward progression of the center of mass during the stance phase of gait. As the mechanical actuator increased the amount of assistance supplied to the center of mass, the magnitude of the largest Lyapunov exponent systematically increased for the hip and ankle joints. These results imply that the performance of the hip and ankle joints during the stance phase may be related to the changes in the nonlinear structure noted in previous investigations [6,7,9]. This is consistent with previous experimental studies where it has been concluded that the stance phase dynamics are dependent on the ankle and hip joints' control properties. The ankle joint supplies a large amount of power for the forward progression of the center of mass [25,26] and the hip joint stabilizes the trunk during the early and late portions of the stance phase [32,33]. However, it cannot be completely concluded if the changes in the nonlinear structure of the hip joint were a result of normal torso control during the stance phase. Since the mechanical horizontal actuator was attached at the waist of the subject, it may have artificially created instabilities in the torso which required an altered control strategy at the hip joint that would not have been present if the center of mass was actuated purely by a toe-off impulse.

The nonlinear structure of the knee joint during the horizontal assistance conditions was not significantly different from normal walking. The lack of clear results for the knee joint may be related to its functional role during gait. The behavior of the knee joint is largely attributed to maintaining the inverted pendulum during stance and limb clearance during the swing [32]. Hence, the knee joint has less influence on the forward progression of the center of mass [14]. However, further inspection of Figure 6 indicates that with the exception of 0%, the knee follows the same increasing linear trend as the ankle and hip joint. Possibly, the knee joint's nonlinear behavior may be also sensitive to the assistive force provided during the stance phase. However, further exploration of this notion is necessary before we can make this conclusion. Possibly, by altering the walking velocity of the subject, the linear trend at the knee joint may be further magnified.

The experiments conducted here were only performed at a medium-high walking velocity. This walking velocity may not be representative of the walking velocity that a disabled subject may select. Since we did not test the influence of horizontal assistance at a wide range of speeds we cannot generalize our results to all populations. Future investigations should explore how the interactive effect of walking speed and forward progression of the center of mass on the nonlinear structure of gait. These insights may lead to new insights on the nature of nonlinear gait patterns and may guide the development of rehabilitative protocols that are aimed at restoring a healthy nonlinear gait.

The passive dynamic walking model was able to predict the changes in the nonlinear structure of human locomotion as the forward progression of the center of mass was assisted. Although this model is highly simplified compared to the human locomotive system, it appears that it provides a well suited template for modeling the control properties of nonlinear gait dynamics. The additions of more life-like properties to this model may prove fruitful for the future research that is directed toward understanding how the neuromuscular properties influence the nonlinear structure of human locomotion. Such simulations and models will provide further insight on what neuromechanical variables influence the nonlinear gait dynamics.

## Conclusion

Horizontal propulsive forces that are applied during the stance phase influence the nonlinear structure of human locomotion. The experimental results presented here infer that the changes in the nonlinear structure may be related to the proper utilization the hip and ankle joint musculature to control the forward progression of the center of mass. Future investigation should determine if the results presented here can be extended to individuals with altered nonlinear gait patterns (*i.e*., elderly, Parkinson's disease). The initial step toward making this connection should be directed towards determining if the results presented here are consistent for different walking speeds. This scientific information will provide further insight on which neuromechanical components that are responsible for changes in the nonlinear structure of gait, and may lead to a better understanding of why the nonlinear gait pattern is altered in pathological populations.

## Competing interests

The author(s) declare that they have no competing interests.

## Authors' contributions

MK conceived of the study and experimental design, carried out the computer simulations, design and fabrication of the horizontal actuator, performed the data collections and processing, and drafted the manuscript. NS participated in the experimental design, interpretation of the results, and drafting of the manuscript. All authors read and approved the final manuscript.
